# Significance of Ki-67 in non-muscle invasive bladder cancer patients: a systematic review and meta-analysis

**DOI:** 10.18632/oncotarget.21899

**Published:** 2017-10-13

**Authors:** Kyungtae Ko, Chang Wook Jeong, Cheol Kwak, Hyeon Hoe Kim, Ja Hyeon Ku

**Affiliations:** ^1^ Department of Urology, Kandong Sacred Heart Hospital, Hallym University College of Medicine, Seoul, Korea; ^2^ Department of Urology, Seoul National University Hospital, Seoul, Korea

**Keywords:** bladder cancer, urothelial carcinoma, Ki-67, prognosis, meta-analysis

## Abstract

**Purpose:**

This meta-analysis evaluated the prognostic significance of Ki-67 in non-muscle invasive bladder cancer (NMIBC).

**Materials and Methods:**

We selected 39 articles including 5,229 patients from Embase, Scopus, and PubMed searches. The primary outcomes, recurrence-free survival (RFS), progression-free survival (PFS), disease-specific survival (DSS), and overall survival (OS) were determined using time-to event hazard ratios (HRs) with 95% confidence intervals (CIs). Study heterogeneity was tested by chi-square and I^2^ statistics. Heterogeneity sources were identified by subgroup meta-regression analysis.

**Results:**

Two studies were prospective; 37 were retrospective. Immunohistochemistry was performed in tissue microarrays or serial sections. A wide range of antibody dilutions and Ki-67 positivity thresholds were used. Study heterogeneity was attributed to analysis results in studies of RFS (*p* < 0.0001). Meta-regression analysis revealed that region and analysis results accounted for heterogeneity in PFS studies (*p* = 0.00471, *p* < 0.0001). High Ki-67 expression was associated with poor RFS (pooled HR, 1.78; 95% CI, 1.48–2.15), poor PFS (pooled HR, 1.28; 95% CI, 1.13–2.15), poor DSS (pooled HR, 2.24; 95% CI, 1.47–2.15), and worse OS (pooled HR, 2.29; 95% CI, 1.24–4.22).

**Conclusions:**

The meta-analysis found that current evidence supports the prognostic value of Ki-67 in NMIBC patients.

## INTRODUCTION

Bladder cancer is the ninth most common cancer worldwide. Approximately 430,000 patients are diagnosed and 165,000 patients die from it annually [1]. Approximately 25% of newly diagnosed cases are muscle invasive bladder cancer (MIBC, ≥ T2), and radical cystectomy is the standard treatment. Other non-muscle invasive bladder cancers (NMIBCs) include stage Ta noninvasive papillary carcinomas and stage T1 tumors that invade the subepithelial connective tissue. The gold standard treatment of NMIBC is transurethral resection of bladder tumor (TURBT) and intravesical Bacillus Calmette–Guérin (BCG) installation. However, 30%–70% of patients experience a recurrence after initial treatment, and 25%–60% progress to MIBC.

As the incidence and survival of bladder cancer increase, the importance of treatment follow-up and predicting the risk of recurrence and progression of individual patients also increases. The outcome of T1 bladder cancer can range from no recurrence to rapid progression to MIBC and metastasis. As progression has a poor prognosis, it is important to distinguish patients who would benefit from early cystectomy and those best managed by bladder-preserving treatments. Currently, such group assignment is challenging. The use of clinical and pathological variables, such as tumor size and number and presence of a carcinoma *in situ* (CIS), to estimate MIBC progression risk has been evaluated [2], but it is difficult to estimate individual prognosis. Characterizing bladder cancer as low or high grade using two-tier criteria of the European Treatment Guidelines or the 2004 World Health Organization classification is difficult, and distinguishing Ta and T1 bladder cancer is problematic because of interobserver error [3]. Tumor markers, such as bcl-2, p53, Ki67, and CK20, are currently under study, but none are in routine clinical use at this time.

Ki-67 is a nuclear protein that is associated with ribosomal RNA transcription and is a marker of cellular proliferation [4]. It is strongly expressed in the growth fraction of cancer cells, and the presence of Ki-67-positive tumor cells indicates a poor survival and recurrence prognosis in prostate and breast cancer and nephroblastoma [5]. Ki-67 has not been confirmed as a poor prognosis marker in NMIBC patients because the reported thresholds of positivity and the immunochemical staining methods vary, making direct comparisons difficult [6]. An expert consensus panel has found that markers, such as Ki-67 and p53, can predict the recurrence and progression of bladder cancer, but the inconsistency of available data indicates their unreliability [7]. This meta-analysis was conducted to increase our understanding of the prognostic significance of Ki-67 in NMIBC patients.

## RESULTS

### Study characteristics

The characteristics of the 39 selected studies are described in Tables 1–3. They were published between 1997 and 2015, 17 were conducted in Asian countries, 17 were conducted in Europe, and five were conducted in America. All but two studies were retrospective, 19 included < 100 patients, 20 included ≥ 100 patients, follow-up ranged from 1 to 267 months, and five studies did not report the duration of follow-up.

**Table 1:** Main characteristics of the eligible studies

Study	Year	Country	Recruit period	Study design	Inclusion and exclusion criteria	Consecutive patients	Definition of outcome
Asakura [20]	1997	Japan	1984–1993	Retrospective	Yes	NA	No
Lee [21]	1997	Korea	1988–1993	Retrospective	Yes	NA	No
Pfister [22]	1999	Canada	1990–1992	Retrospective	Yes	NA	No
Tomobe [23]	1999	Japan	1989–1994	Retrospective	No	NA	No
Wu [24]	2000	Taiwan	1990–1997	Retrospective	Yes	NA	No
Blanchet [25]	2001	France	1989–1990	Prospective	No	Yes	Yes
Kamai [26]	2001	Japan	1987–1997	Retrospective	No	Yes	No
Kilicli-Camur [27]	2002	Turkey	NA	Retrospective	No	NA	Yes
Sgambato [28]	2002	Italy	1990–1995	Retrospective	Yes	Yes	Yes
Yan [29]	2002	USA	1994–1999	Retrospective	Yes	Yes	No
Dybowski [30]	2003	Poland	1994–1995	Retrospective	Yes	NA	No
Santos [31]	2003	Portugal	1989–1996	Retrospective	Yes	Yes	Yes
Su [32]	2003	Japan	NA	Retrospective	No	NA	Yes
Mhawech [33]	2004	Switzerland	1997–2000	Retrospective	Yes	NA	Yes
Krüger [34]	2005	Germany	1987–1999	Retrospective	Yes	Yes	Yes
Theodoropoulos [35]	2005	Greece	1993–2003	Retrospective	Yes	No	Yes
Gonzalez-Campora [36]	2006	Spain	1991–1997	Retrospective	No	Yes	Yes
Quintero [37]	2006	Spain	1990–1994	Retrospective	No	Yes	Yes
Yin [38]	2006	China	NA	Retrospective	No	Yes	No
Maeng [39]	2010	Korea	2001–2007	Retrospective	No	NA	No
Miyake [40]	2010	Japan	2000–2005	Retrospective	No	Yes	No
Seo [41]	2010	Korea	2001–2007	Retrospective	Yes	NA	Yes
van Rhijn [10]	2010	Netherlands	NA	Retrospective	No	NA	Yes
Behnsawy [42]	2011	Japan	2000–2007	Retrospective	No	Yes	No
Wosnitzer [43]	2011	USA	NA	Retrospective	No	NA	No
Acikalin [6]	2012	Turkey	1996–2007	Retrospective	No	NA	Yes
Chen [11]	2012	China	NA	Retrospective	No	NA	Yes
Ogata [44]	2012	Brazil	2005–2010	Retrospective	Yes	NA	No
Oderda [45]	2013	Italy	1994–2004	Prospective	No	NA	Yes
Okazoe [46]	2013	Japan	2006–2009	Retrospective	No	NA	No
Park [47]	2013	Korea	1990–2007	Retrospective	No	NA	Yes
Ruan [48]	2013	China	2007–2010	Retrospective	Yes	NA	No
Ben Abdelkrim [14]	2014	Tunisia	2001–2003	Retrospective	No	NA	Yes
Bertz [18]	2014	Germany	1989–2006	Retrospective	No	NA	No
Ding [15]	2014	China	2000–2010	Retrospective	No	NA	Yes
Mangrud [49]	2014	Norway	2002–2006	Retrospective	Yes	Yes	Yes
Pan [50]	2014	Taiwan	1991–2005	Retrospective	No	NA	Yes
Özyalvaçli [16]	2015	Turkey	2005–2013	Retrospective	No	Yes	Yes
Poyet [17]	2015	Switzerland	1990–2006	Retrospective	No	Yes	Yes

NA: not available.

**Table 2:** Patient characteristics of the eligible studies

Study	No. of patients	Median age, range (years)	Gender (male/female)	Intravesical therapy (no.)	Median follow-up, range (months)
Asakura [20]	104	63 (mean), 28–90	78/26	Chemotherapy (6)	42 (mean), 3–134
Lee [21]	32	NA, 30–81	28/4	BCG (32)	NA
Pfister [22]	244	65.1 (mean), NA	NA	No	47 (mean), NA
Tomobe [23]	50	63.9 (mean), 22–88	43/7	Chemotherapy or BCG (32)	44 (mean), 5–80
Wu [24]	86	NA	NA	NA	NA
Blanchet [25]	70	62.6 (mean), 21–84	66/4	BCG (57)	64, 12–111
Kamai [26]	86	NA	NA	MMC, doxorubicin or BCG (NA)	50, 3–124
Kilicli-Camur [27]	118	60.2 (mean), 29–86	NA	NA	31.4 (mean), 24–60
Sgambato [28]	96	68 (mean), 29–92	83/13	BCG (NA)	50 (mean), 24–102
Yan [29]	270	71 (mean), NA	196/71, unknown (3)	BCG (66)	19, (1–54)
Dybowski [30]	45	NA	NA	NA	64, 1–82
Santos [31]	159	66, 21–88	115/44	Chemotherapy (65), BCG (17)	46.5, 4–123
Su [32]	79	64, 34–91	66/13	MMC or Adriamycin (74)	48.7 (mean), 4–78
Mhawech [33]	49	70.3 (mean), 52–90	44/5	BCG (7)	12, 3–77
Krüger [34]	73	68, NA	60/13	BCG (73)	NA
Theodoropoulos [35]	140	69, 23–89	107/33	Epirubicin or BCG (114)	41, 8–131
Gonzalez-Campora [36]	147	66 (mean), 30–95	127/20	BCG (NA)	75 (mean), 5–12 yr
Quintero [37]	164	61 (mean), 29–93	143/21	BCG (NA)	75, 60–144
Yin [38]	101	NA	81/20	BCG (101)	54, 20–68.6 (10–90% percentiles)
Maeng [39]	55	67 (mean), 33–84	40/15	NA	26.2 (mean), 3–70
Miyake [40]	109	68.5 (mea), 36–94	19/14	Anthracycline (16), doxorubicin (1), epirubicin (13), pirarubicin (2), BCG (19)	48, 1–99
Seo [41]	129	64.2 (38–88)	104/25	MMC (129)	48.6 (mean), 6.1–96
van Rhijn [10]	230	65.1 (mean), NA	175/55	NA	8.6 yr, 6.6–11.3 yr (IQR)
Behnsawy [42]	161	NA	137/24	Unknown regimen (49)	47, 13–93
Wosnitzer [43]	32	70.3, 44–89	25/7	Docetaxel (17), nanoparticle albumin-bound docetaxel (15)	22, 11–75
Acikalin [6]	68	63, 35–85	66/2	NA	51, 12–132
Chen [11]	72	61.3 (mean), 27–87	58/14	MMC, epirubicin, pirarubicin (NA)	63.4 (mean), 16–93
Ogata [44]	43	70, 39–85	35/8	NA	NA, 12–71
Oderda [45]	192	73.2 (mean), NA	166/26	BCG (192)	100, 2–229
Okazoe [46]	71	72, 41–95	59/12	Unknown regimen (31)	9.8, 1.0–51.8
Park [47]	70	66, 31–85	53/8	BCG (70)	60, 6–217
Ruan [48]	126	64.5 (mean), 29–90	103/23	NA	NA
Ben Abdelkrim [14]	71	63.1 (mean), 39–88	67/4	NA	28, 3–77
Bertz [18]	309	71.7, 38–87	237/72	BCG (309)	49, 5–172
Ding [15]	332	67, 21–92	273/59	NA	47, 2–124
Mangrud [49]	193	74, 39–95	148/45	BCG (NA)	75, 1–127
Pan [50]	605	71 (mean), 23–92	511/94	MMC (272), doxorubicin (67), epirubicin (130), BCG (132)	NA
Özyalvaçli [16]	90	NA	83/7	NA	32.8, 36.2–103.6 (IQR)
Poyet [17]	158	69.5, 32–92	131/43	NA	110.6, 32.4–266.8

NA: not available, BCG: bacille Calmette-Guérin, MMC: mitomycin C, IQR: interquartile range.

**Table 3:** Tumor characteristics of the eligible studies

Study	T stage			Grade			Concomitant CIS		Multiplicity		Size		Tumor architecture		History	
	Tis	Ta	T1	G1	G2	G3	Absent	Present	Single	Multiple	< 3 cm	≥ 3 cm	Papillary	Non-papillary	Primary	Recurrent
Asakura [20]		61	43	30	63	11	NA	NA	NA	NA	NA	NA	NA	NA	104	NA
Lee [21]	0	0	42	0	16	16	30	2	42	0	NA	NA	26	6	17	15
Pfister [22]	0	194	50	83	NA	NA	NA	NA	163	81	152	92	NA	NA	244	0
Tomobe [23]	0	6	44	15	28	7	NA	NA	26	24	NA	NA	NA	NA	34	16
Wu [24]	NA	NA	NA	NA	NA	0	NA	NA	86	0	NA	NA	86	0	86	0
Blanchet [25]	0	43	27	12	25	33	63	7	30	17	NA	NA	NA	NA	70	0
Kamai [26]	NA	NA	NA	NA	NA	NA	NA	NA	NA	NA	NA	NA	NA	NA	NA	NA
Kilicli-Camur [27]	0	59	59	45	51	22	NA	NA	NA	NA	NA	NA	NA	NA	60	58
Sgambato [28]	0	42	54	13	51	32	NA	NA	96	0	NA	NA	NA	NA	96	0
Yan [29]	0	215	55	57	183	30	270	0	NA	NA	NA	NA	NA	NA	NA	NA
Dybowski [30]	0	25	20	NA	NA	NA	45	0	NA	NA	NA	NA	NA	NA	NA	NA
Santos [31]	0	56	103	61	98	0	159	0	122	37	NA	NA	NA	NA	159	0
Su [32]	0	33	46	23	56	0	NA	NA	43	36	65	14	56	23	79	0
Mhawech [33]	0	0	49	0	38	11	NA	NA	30	19	NA	NA	NA	NA	49	0
Krüger [34]	0	0	73	0	33	40	NA	NA	27	46	NA	NA	NA	NA	73	0
Theodoropoulos [35]	0	42	98	30	88	22	NA	NA	NA	NA	NA	NA	NA	NA	140	0
Gonzalez-Campora [36]^*^	0	63	84	29	92	26	NA	NA	NA	NA	57	90	NA	NA	147	0
Quintero [37]^*^	0	80	84	31	92	41	NA	NA	NA	NA	109	55	NA	NA	164	0
Yin [38]^*^	0	54	47	0	59	42	101	0	NA	NA	NA	NA	NA	NA	NA	NA
Maeng [39]^*^	0	38	17	10	22	23	NA	NA	44	11	35	20	50	5	33	22
Miyake [40]	2	24	83	9	74	26	98	11	54	55	87	22	NA	NA	109	0
Seo [41]	0	81	46	31	76	22	129	0	36	84	60	57	104	15	101	28
van Rhijn [10]	0	171	59	88	108	34	218	12	165	65	NA	NA	NA	NA	230	0
Behnsawy [42]	0	65	25	29	49	12	76	14	46	44	72	18	80	10	90	0
Wosnitzer [43]^*^	9	5	18	0	0	32	24	8	NA	NA	NA	NA	NA	NA	0	32
Acikalin [6]	0	0	68	11	31	26	NA	NA	23	45	16	52	NA	NA	NA	NA
Chen [11]^*^	0	19	53	16	38	18	NA	NA	49	23	43	29	NA	NA	NA	NA
Ogata [44]^*^	0	41	2	0	26	14	43	0	43	0	24	19	43	0	43	0
Oderda [45]	0	121	115	53	76	63	182	10	58	134	159	31	NA	NA	113	79
Okazoe [46]^*^	2	53	16	0	46	25	NA	NA	34	37	54	12	58	13	44	27
Park [47]	0	0	61	0	0	61	56	5	23	38	36	25	38	23	61	0
Ruan [48]	0	0	126	NA	NA	55	126	0	75	51	NA	NA	NA	NA	NA	NA
Ben Abdelkrim [14]	0	39	32	26	35	10	NA	NA	NA	NA	NA	NA	NA	NA	71	0
Bertz [18]	0	0	309	0	89	220	202	106	106	203	128	181	257	52	NA	NA
Ding [15]	0	204	128	114	168	50	309	23	NA	NA	221	111	NA	NA	NA	NA
Mangrud [49]	0	154	39	44	98	51	171	22	92	73	NA	NA	NA	NA	193	0
Pan [50]^*^	0	336	231	38	256	311	NA	NA	NA	NA	NA	NA	NA	NA	NA	NA
Özyalvaçli [16]^*^	0	41	49	0	45	45	NA	NA	53	37	46	43	NA	NA	90	0
Poyet [17]	0	90	68	44	86	28	12	146	115	43	NA	NA	151	7	158	0

^*^Grading according to the 2004 WHO classification system: papillary urothelial neoplasm of low malignant potential, low grade and high grade.

CIS: carcinoma *in situ*, NA: not available.

### Immunohistochemistry

Immunohistochemistry (IHC) was performed using tissue microarrays of 1–2 mm diameter samples of representative tissues and using slide mounted serial tissue sections in the other 34 studies. Fifteen of the 39 studies evaluated IHC staining in formalin-fixed paraffin-embedded tissue blocks, but did not identify the primary antibody used, and a wide range of antibody dilutions was reported (1/20 to 1/200). In 33 studies, immunopositivity was defined by the presence of nuclear staining, but the cutoff percentage for positive or negative expression (% IHC cutoff) and the reported percentage of Ki-67-positive cells varied widely among studies. Twenty studies reported blinded evaluation of Ki-67 expression (Table 4).

**Table 4:** Immunohistochemical analysis of the eligible studies

Study	Tissue section	Primary antibody	Dilution	Compartment	Definition of ki-67 index	% IHC cut-off	% ki-67 positive	Interpretation
Asakura [20]	All specimens	NA	1:200	Nuclei	Yes	5.35	50	NA
Lee [21]	All specimens	NA	NA	Nuclei	Yes	16	50	Blind
Pfister [22]	All specimens	Monoclonal	1:50	Nuclei	No	10	70	Blind
Tomobe [23]	All specimens	NA	1:200	Nuclei	Yes	15.5	50	NA
Wu [24]	All specimens	NA	1:100	Nuclei	Yes	10.9	50	Blind
Blanchet [25]	All specimens	Monoclonal	NA	NA	Yes	13	18.5	Blind
Kamai [26]	All specimens	Monoclonal	NA	Nuclei	Yes	30	18.6	NA
Kilicli-Camur [27]	All specimens	Monoclonal	1:30	Nuclei	Yes	25	NA	NA
Sgambato [28]	All specimens	Monoclonal	1:100	Nuclei	Yes	10	65.6	Blind
Yan [29]	All specimens	NA	NA	Nuclei	No	25	34.2	NA
Dybowski [30]	All specimens	Monoclonal	1:50	Nuclei	No	30	50	Blind
Santos [31]	All specimens	NA	1:50	Nuclei	Yes	18	50	NA
Su [32]	All specimens	NA	1:50	Nuclei	Yes	18	50	NA
Mhawech [33]	TM (1.6 mm core)	NA	1:50	Nuclei	Yes	NA	50	Blind
Krüger [34]	TM (2 × 2 mm)	Monoclonal	1:20	Nuclei	Yes	Continuous	-	Blind
Theodoropoulos [35]	All specimens	NA	Prediluted	Nuclei	Yes	8.6	50	Blind
Gonzalez-Campora [36]	All specimens	Monoclonal	1:20	Nuclei	Yes	10	18.4	NA
Quintero [37]	All specimens	Monoclonal	Prediluted	Nuclei	Yes	13	10.4	NA
Yin [38]	All specimens	Monoclonal	1:100	Nuclei	Yes	20	24.8	NA
Maeng [39]	All specimens	NA	1:80	Nuclei	Yes	25	36.4	NA
Miyake [40]	All specimens	Monoclonal	Prediluted	Nuclei	Yes	25	40.4	Blind
Seo [41]	All specimens	Monoclonal	1:50	Nuclei	Yes	25	36.4	NA
van Rhijn [10]	All specimens	NA	NA	NA	NA	25	NA	Blind
Behnsawy [42]	All specimens	Monoclonal	1:200	Nuclei	Yes	5	28.6	Blind
Wosnitzer [43]	All specimens	Monoclonal	NA	NA	Yes	10	50	Blind
Acikalin [6]	All specimens	Monoclonal	1:50	Nuclei	Yes	10	69.1	Blind
Chen [11]	All specimens	Monoclonal	1:50	Nuclei	Yes	25	47.2	NA
Ogata [44]	All specimens	Monoclonal	1:100	NA	No	20	58.1	NA
Oderda [45]	All specimens	Monoclonal	1:10	Nuclei	Yes	20	NA	NA
Okazoe [46]	All specimens	Monoclonal	1:100	Nuclei	Yes	18	29.6	Blind
Park [47]	TM (1 mm core)	Monoclonal	1:200	Nuclei	Yes	10.4	40	Blind
Ruan [48]	All specimens	Polyclonal	1:50	Nuclei	Yes	10	55.6	Blind
Ben Abdelkrim [14]	All specimens	NA	1:50	Nuclei	Yes	10	38	Blind
Bertz [18]	All specimens	Monoclonal	1:50	Nuclei	Yes	15	64.4	NA
Ding [15]	All specimens	Monoclonal	1:100	Nuclei	No	25	32.5	NA
Mangrud [49]	All specimens	NA	NA	NA	Yes	39	25	NA
Pan [50]	TM (2 mm core)	NA	1:100	Nuclei	Yes	20/80	NA	Blind
Özyalvaçli [16]	All specimens	Monoclonal	NA	Nuclei	Yes	10	27.8	Blind
Poyet [17]	TM (1 mm core)	NA	1:50	NA	Yes	10	38.4	NA

IHC: immunohistochemistry, NA: not available, TM: tissue microarray.

### Study outcomes

**Figure 1:**
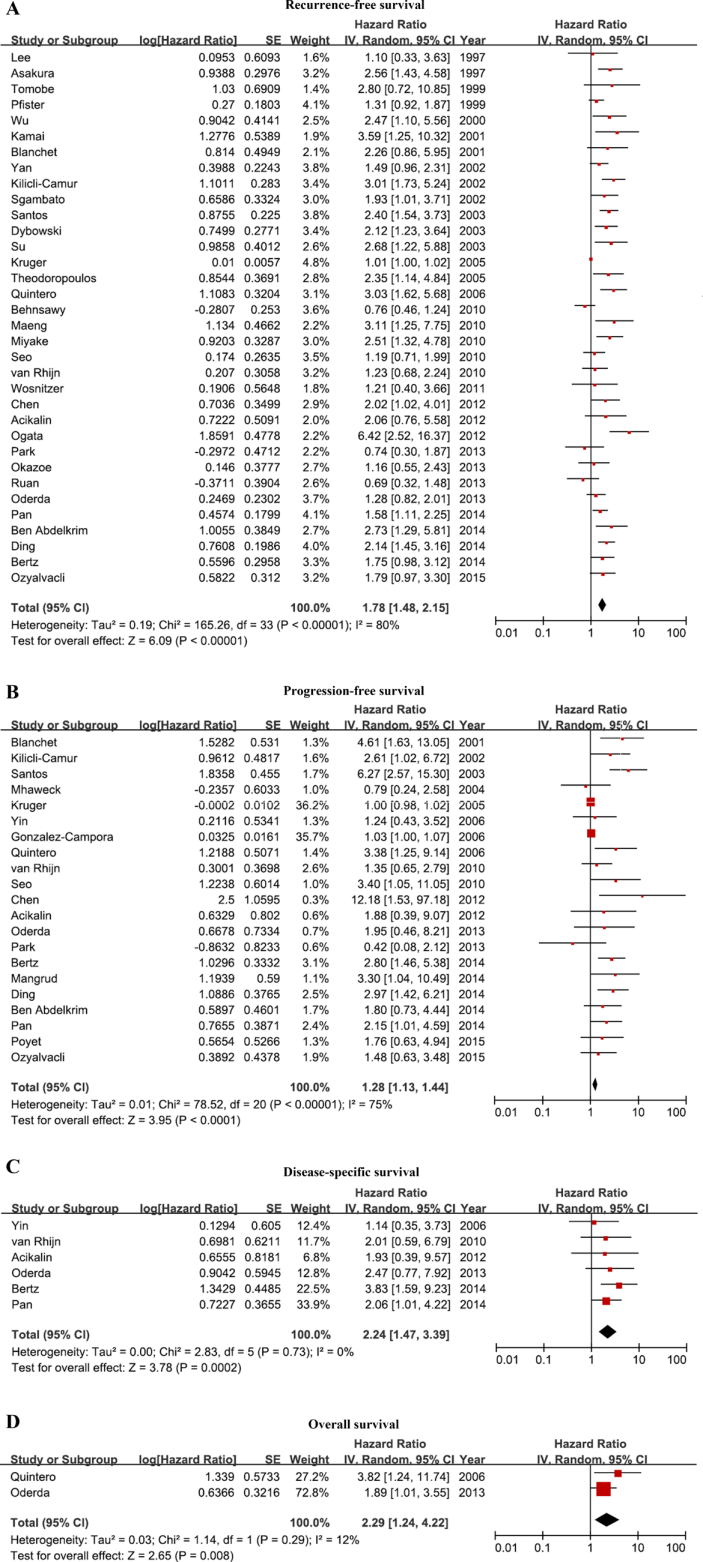
Forest plots of the hazard ratios High Ki-67 expression indicated poor bladder cancer prognosis. (**A**) Recurrence-free survival, (**B**) progression-free survival, (**C**) disease-specific survival, (**D**) overall survival. Between-study heterogeneity was detected in the effect of Ki-67 expression on RFS and PFS.

Of the 39 studies, the association of Ki-67 expression with recurrence-free survival (RFS) was reported in 34 (4,581 patients), with progression-free survival (PFS) in 21 (3,400 patients), with disease-specific survival (DSS) in six (1,505 patients), and with overall survival (OS) in two (356 patients) studies (Tables 5–8). The most common cofactors included in the multivariate analysis of the risk of outcome were grade and T stage. Forest plots of the hazard ratios (HRs) reported in individual studies and those from the meta-analysis are shown in Figure 1. Despite the use of strict inclusion criteria, between-study heterogeneity was detected in the effect of Ki-67 expression on RFS and PFS, with *p* <0.05 and I^2^ ≥ 50%.

**Table 5:** Estimation of the hazard ratio for recurrence-free survival

Study	Analysis	HR estimation	Co-factors	Analysis results
Asakura [20]	Multivariate	HR, 95% CI	T stage, grade, multiplicity, size	Significant
Lee [21]	Multivariate	HR, 95% CI	P53, bcl-2, cathepsin-D	Not significant
Pfister [22]	Multivariate	HR, 95% CI	T stage, grade, multiplicity, size, p53, MDM2, p21	Not significant
Tomobe [23]	Multivariate	HR, *p* value	T stage, grade, multiplicity, size, recurrence history, whole NOR, proliferating NOR, resting NOR	Not significant
Wu [24]	Multivariate	HR, 95% CI	T stage, grade, p53, bcl-2	Significant
Blanchet [25]	Univariate	Event no., *P* value	-	Not significant
Kamai [26]	Multivariate	HR, 95% CI	Grade, p27, cyclin E	Significant
Kilicli-Camur [27]	Univariate	Event no., *P* value	-	Significant
Sgambato [28]	Multivariate	HR, 95% CI	Age, T stage, grade, p27, cyclin D1	Significant
Yan [29]	Multivariate	HR, 95% CI	T stage, p53	Not significant
Dybowski [30]	Univariate	Event no., *P* value	-	Significant
Santos [31]	Multivariate	HR, 95% CI	T stage, grade, multiplicity, BCG, p53	Significant
Su [32]	Multivariate	HR, 95% CI	T stage, tumor architecture, p53, c-erbB-2	Significant
Krüger [34]	Multivariate	HR, 95% CI	Grade, p53	Not significant
Theodoropoulos [35]	Multivariate	HR, 95% CI	T stage, grade, apoptotic index, p53, bcl-2, VEGF, MVD, HIF-1α	Significant
Quintero [37]	Multivariate	HR, 95% CI	Size	Significant
Maeng [39]	Univariate	HR, 95% CI	-	Significant
Miyake [40]	Multivariate	HR, 95% CI	Grade, p53, HO-1	Significant
Seo [41]	Univariate	HR, 95% CI	-	Not significant
van Rhijn [10]	Multivariate	HR, 95% CI	Age, sex, hospital, T stage, grade, concomitant CIS, multiplicity, size, EORTC risk score, *FGFR3*	Not significant
Behnsawy [42]	Univariate	HR, 95% CI	-	Not significant
Wosnitzer [43]	Multivariate	HR, 95% CI	Age, sex, T stage, concomitant CIS, p53, stathmin, tau	Not significant
Acikalin [6]	Multivariate	HR, 95% CI	Age, grade, size, multiplicity, mapsin	Not significant
Chen [11]	Multivariate	HR, 95% CI	Age, sex, T stage, grade, multiplicity, size, intravesical instillation, VEGF	Significant
Ogata [44]	Univariate	Event no., *P* value	-	Significant
Oderda [45]	Multivariate	HR, 95% CI	Age, T stage, grade, ,multiplicity, size, p53	Not significant
Okazoe [46]	Univariate	HR, 95% CI	-	Not significant
Park [47]	Multivariate	HR, 95% CI	p53, pRb, PTEN, p27, *FGFR3*, CD9	Not significant
Ruan [48]	Multivariate	HR, 95% CI	Age, sex, grade, multiplicity, size, Sox2	Significant
Ben Abdelkrim [14]	Univariate	Event no., *P* value	-	Significant
Bertz [18]	Multivariate	HR, 95% CI	Age, sex, grade, concomitant CIS, tumor architecture, p53, CK20	Not significant
Ding [15]	Multivariate	HR, 95% CI	T stage, grade, concomitan CIS, multiplicity, size	Significant
Pan [50]	Multivariate	HR, 95% CI	T stage, grade, multiplicity, size, intravesical instillation, p53, HSP27, COX2, cyclin D1, p16, pRb, p27, p21, EGFR, E-cadherin, EpCam, no. of altered markers	Significant
Özyalvaçli [16]	Multivariate	HR, 95% CI	T stage, smoking, size, P16d	Not significant

HR: hazard ratio, CI: confidence interval, NOR: nucleolar organizer regions, BCG: bacille Calmette-Guérin, VEGF: vascular endothelial growth factor, MVD, microvessel density, HIF: hypoxia-inducible factor, CIS: carcinoma *in situ*, EORTC: European Organization for Research and Treatment of Cancer, EGFR: epithelial growth factor receptor.

**Table 6:** Estimation of the hazard ratio for progression-free survival

Study	Analysis	HR estimation	Co-factors	Analysis results
Blanchet [25]	Multivariate	HR, 95% CI	T state, grade, concomitant CIS, multiplicity, size	Significant
Kilicli-Camur [27]	Univariate	Event no., *P* value	-	Significant
Santos [31]	Multivariate	HR, 95% CI	T stage, grade, multiplicity. BCG, p53	Significant
Mhawech [33]	Multivariate	HR, 95% CI	P53, p21, cyclin D1, p27, p16	Not significant
Krüger [34]	Univariate	HR, 95% CI	-	Not significant
Gonzalez-Campora [36]	Multivariate	HR, 95% CI	NA	Significant
Quintero [37]	Multivariate	HR, 95% CI	None	Significant
Yin [38]	Multivariate	HR, 95% CI	Age, T stage, grade, BIRC5-cytoplasmic labeling index, , BIRC5-nuclear labeling index	Not significant
Seo [41]	Multivariate	HR, 95% CI	T stage, grade, tumor architecture, lymphovascular invasion	Significant
van Rhijn [10]	Multivariate	HR, 95% CI	Age, sex, hospital, T stage, grade, concomitant CIS, multiplicity, size, EORTC risk score, *FGFR3*	Not significant
Acikalin [6]	Multivariate	HR, 95% CI	Age, grade, size, multiplicity, mapsin	Not significant
Chen [11]	Multivariate	HR, 95% CI	Age, sex, T stage, grade, multiplicity, size, intravesical instillation, VEGF	Significant
Oderda [45]	Multivariate	HR, 95% CI	Age, T stage, grade, ,multiplicity, size, p53	Not significant
Park [47]	Multivariate	HR, 95% CI	p53, pRb, PTEN, p27, *FGFR3*, CD9	Not significant
Ben Abdelkrim [14]	Univariate	Event no., *P* value	-	Not significant
Bertz [18]	Multivariate	HR, 95% CI	Age, sex, grade, concomitant CIS, tumor architecture, p53, CK20	Significant
Ding [15]	Multivariate	HR, 95% CI	T stage, grade, concomitan CIS, multiplicity, size	Significant
Mangrud [49]	Univariate	HR, 95% CI	-	Significant
Pan [50]	Multivariate	HR, 95% CI	T stage, grade, multiplicity, size, intravesical instillation, p53, HSP27, COX2, cyclin D1, p16, pRb, p27, p21, EGFR, E-cadherin, EpCam, no. of altered markers	Significant
Özyalvaçli [16]	Univariate	Event no., *P* value	-	Not significant
Poyet [17]	Multivariate	HR, 95% CI	Grade, tumor architecture, Cx43	Not significant

HR: hazard ratio, CI: confidence interval, CIS: carcinoma *in situ*, BCG: bacille Calmette-Guérin, NA: not available, EORTC: European Organization for Research and Treatment of Cancer, VEGF: vascular endothelial growth factor, EGFR: epithelial growth factor receptor.

**Table 7:** Estimation of the hazard ratio for disease-specific survival

Study	Analysis	HR estimation	Co-factors	Analysis results
Yin [38]	Multivariate	HR, 95% CI	Age, T stage, grade, BIRC5-cytoplasmic labeling index, , BIRC5-nuclear labeling index	Not significant
van Rhijn [10]	Multivariate	HR, 95% CI	Age, sex, hospital, T stage, grade, concomitant CIS, multiplicity, size, EORTC risk score, *FGFR3*	Not significant
Acikalin [6]	Univariate	Event no., *P* value	-	Not significant
Oderda [45]	Multivariate	HR, 95% CI	Age, T stage, grade, ,multiplicity, size, p53	Not significant
Bertz [18]	Multivariate	HR, 95% CI	Age, sex, grade, concomitant CIS, tumor architecture, p53, CK20	Significant
Pan [50]	Multivariate	HR, 95% CI	T stage, grade, multiplicity, size, intravesical instillation, p53, HSP27, COX2, cyclin D1, p16, pRb, p27, p21, EGFR, E-cadherin, EpCam, no. of altered markers	Significant

HR: hazard ratio, CI: confidence interval, CIS: carcinoma *in situ*, EORTC: European Organization for Research and Treatment of Cancer, EGFR: epithelial growth factor receptor.

**Table 8:** Estimation of the hazard ratio for overall survival

Study	Analysis	HR estimation	Co-factors	Analysis results
Quintero [37]	Multivariate	HR, 95% CI	Size, p27	Significant
Oderda [45]	Multivariate	HR, 95% CI	Age, T stage, grade, ,multiplicity, size, p53	Significant

HR: hazard ratio, CI: confidence interval.

### RFS

Overall, the pooled HR for RFS in 34 studies was 1.78 (95% CI, 1.48–2.15), suggesting that high Ki-67 expression indicated poor bladder cancer prognosis. However, significant heterogeneity was observed in the studies (I^2^ = 80%, *p* < 0.00001) (Figure 1A). Subgroup meta-regression by publication year, region, number of patients, HR estimation, and analysis results identified analysis results as the only possible explanation for heterogeneity (*p* < 0.0001, Table 9). The other variables in the subgroup analyses did not include any heterogeneity of data.

**Table 9:** Subgroup analysis for recurrence-free survival

	No. of included articles	No. of cases	Pooled HR (95% CI)	Chi^2^ (*p* value)	I^2^	*P*_h_^*^
Publication year						0.1633
1997–2009	16	1,816	2.05 (1.52–2.76)	92.96 (< 0.00001)	84%	
2010–2015	18	2,765	1.58 (1.26–1.96)	37.18 (0.003)	54%	
Region						0.7686
Asia	16	2,167	1.66 (1.29–2.13)	33.06 (0.005)	55%	
Europe	14	1,825	1.91 (1.41–2.58)	76.87 (< 0.00001)	83%	
America	4	589	1.81 (1.04–3.15)	9.93 (0.02)	70%	
No. of patients						0.3895
< 100	18	1,189	1.95 (1.44–2.65)	69.11 (< 0.00001)	75%	
≥ 100	16	3,392	1.66 (1.36–2.03)	37.44 (0.001)	60%	
HR estimation						0.5542
Univariate	9	763	1.99 (1.30–3.05)	29.03 (0.0003)	72%	
Multivariate	25	3,818	1.72 (1.40–2.12)	111.81 (< 0.00001)	79%	
Analysis results						< 0.0001
Not significant	16	2,091	1.22 (1.05–1.43)	22.48 (0.10)	33%	
Significant	18	2,490	2.28 (1.93–2.70)	22.27 (0.17)	24%	

HR: hazard ratio, CI: confidence interval.

P_h_^*^ for heterogeneity between subgroups with meta-regression analysis.

## PFS

A meta-analysis of 21 studies found that high Ki-67 expression was significantly associated with poor PFS (pooled HR, 1.28; 95% CI, 1.13–1.44). However, the Cochrane *Q* test (*p* < 0.00001) and an I^2^ = 75% could not exclude significant heterogeneity (Figure 1B). Meta-regression analysis revealed that region accounted for part of the study heterogeneity for PFS (*p* = 0.00471, Table 10). In addition, analysis results was found to significantly affect the relationship between Ki-67 expression and PFS (*p* < 0.0001). Other variables included in this subgroup analysis did not include any heterogeneity of data.

**Table 10:** Subgroup analysis for progression-free survival

	No. of included articles	No. of cases	Pooled HR (95% CI)	Chi^2^ (*p* value)	I^2^	*P*_h_^*^
Publication year						0.1633
1997–2009	8	881	1.08 (0.97–1.19)	37.11 (< 0.00001)	81%	
2010–2015	13	2,519	2.11 (1.62–2.75)	11.71 (0.47)	0%	
Region						0.0471
Asia	6	1,309	2.16 (1.19–3.93)	8.96 (0.11)	44%	
Europe	15	2,091	1.17 (1.05–1.30)	55.75 (< 0.00001)	75%	
No. of patients						0.2529
< 100	8	563	1.53 (0.91–2.59)	18.15 (0.01)	61%	
≥ 100	13	2,837	2.26 (1.50–3.43)	54.85 (< 0.00001)	78%	
HR estimation						0.418
Univariate	5	545	1.61 (0.97–2.69)	10.50 (0.03)	62%	
Multivariate	16	2,855	2.11 (1.41–3.15)	62.59 (< 0.00001)	76%	
Analysis results						< 0.0001
Not significant	10	1,102	1.00 (0.98–1.02)	7.10 (0.63)	0%	
Significant	11	2,298	3.02 (1769–5.21)	66.75 (< 0.00001)	85%	

HR: hazard ratio, CI: confidence interval, NMIBC: non-muscle invasive bladder cancer.

P_h_^*^ for heterogeneity between subgroups with meta-regression analysis.

## DSS

A meta-analysis of six studies found that high Ki-67 expression was significantly associated with poor DSS (pooled HR, 2.24; 95% CI, 1.47–3.39). No significant study heterogeneity was found (I^2^ = 0%, *p* = 0.73; Figure 1C).

## OS

Meta-analysis of the two studies evaluating the association of ki-67 expression with OS found that a high Ki-67 expression predicted a worse outcome, with a pooled HR of 2.29 (95% CI, 1.24–4.22). Inter-study heterogeneity was not significant (I^2^ = 12%, *p* = 0.29) (Figure 1D).

### Sensitivity analysis

One-way sensitivity analyses were conducted by stepwise exclusion of single studies and recalculating the pooled HR for the remaining studies. No significant differences were observed among the results obtained at each step of the analysis (data not shown), demonstrating that the overall results of the meta-analysis were statistically reliable.

### Publication bias

**Figure 2:**
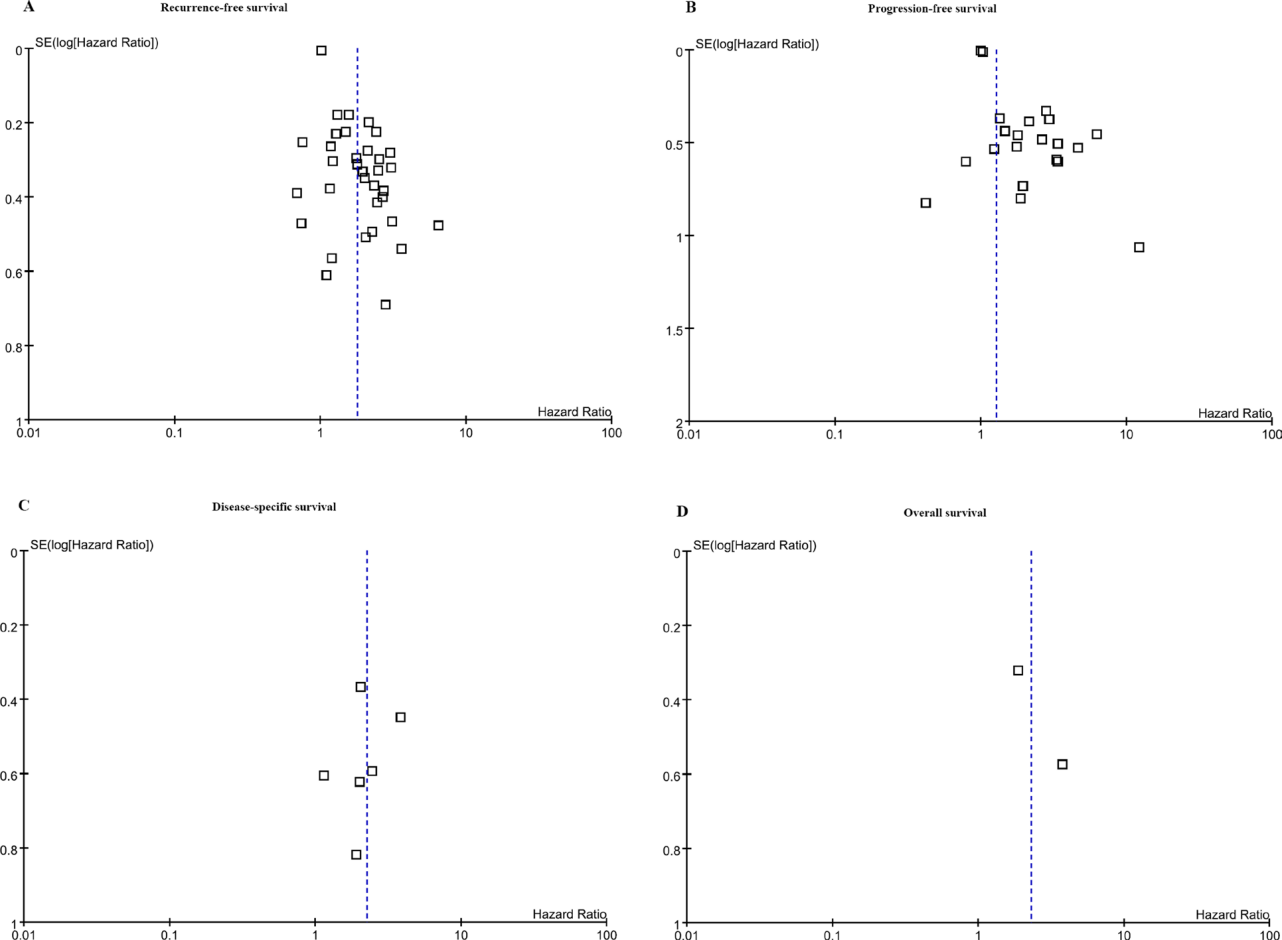
Begg tests for (**A**) recurrence-free survival and (**B**) progression-free survival confirmed the funnel plot symmetry and lack of evidence of publication bias. Fewer than 10 studies were included in meta-analyses of (**C**) disease-specific survival and (**D**) overall survival.

Because fewer than 10 studies were included in meta-analyses of DSS and OS, it was not reasonable to estimate the potential for publication bias. No obvious asymmetry was evident in any of the funnel plots shown in Figure 2. The *p*-values of the Begg tests for RFS and PFS were > 0.05 (*p* = 0.4676 for RFS and 0.4324 for PFS), which confirmed the funnel plot symmetry and lack of evidence of publication bias.

## DISCUSSION

About 75% of newly diagnosed bladder cancers are NMIBC localized in the subepithelial connective tissue [8]. After initial TURBT, NMIBC patients undergo cystoscopy every 3 months for the first year to monitor recurrence and progression. This protocol is painful and is also a financial burden; however, because progression to MIBC has a bad prognosis for the patients, ongoing cystoscopy and radiological evaluation are required. Early cystectomy for high risk T1 bladder cancer patients who are expected to progress is important because it can increase survival. On the other hand, radical cystectomy is a surgical procedure with many complications and requires use of urostomy bags or clean intermittent catheterizations, both of which have negative effects on daily activities. Efforts to distinguish candidates for early cystectomy or bladder preservation are complicated by the heterogeneous clinical behavior of bladder cancer.

Until recently, predicting the progression from NMIBC to MIBC has relied on clinicopathological variables, such as tumor size, grade, multiplicity, and diagnosis of CIS. However, even in cases of the same stage and grade of NMIBC, the clinical course can vary from no recurrence to rapid progression, making it difficult to predict the course. In addition, inter-pathologist variation in interpretation of TURBT specimens can occur because of malorientation, cautery artifacts, and other reasons. Given the current situation, reliable molecular markers would assist in making clinical decisions.

Previous studies of tumorigenesis indicated that changes at the molecular level precede changes in cellular morphology [9]. Changes in gene expression in multiple molecular pathways have been related to the development of bladder cancer. Ki-67 has been associated with expression of oncogenes or tumor suppressor genes, such as Connexin 43, Sox2, G protein-coupled receptor 87, heme oxygenase-1, p53, and p27 [17, 26, 37, 40, 46, 48]. IHC assays of proliferation markers, such as the Ki-67 and fibroblast growth factor receptor (FGFR)-3 are available in most pathology laboratories and have high reproducibility [10, 11]. IHC is currently used worldwide by over 90% of pathologists to diagnose bladder cancer, and Ki-67 is already used as a prognostic marker in over 84% of specimens in Europe [12]. Another advantage of this biologic marker is that objective measurements are possible and changes in expression can be compared after the therapeutic intervention.

Despite many advantages, biologic markers are not widely used to make clinical decisions because difficulties in making direct comparisons of study results have resulted in lack of consensus on their usefulness. In this meta-analysis, the overexpression threshold varied from 5% to 25% and the variation in positive Ki-67 expression was from 10% to 70 percent. Reasons for the inconsistency of previous study results include different follow-up protocols after TURBT, and differences in patient ethnicity, geography, tumor stage, tissue sectioning methods, and the primary antibodies and antibody dilutions used in each study [6]. The importance of these differences was apparent in the inter-study heterogeneity detected in the meta-analysis, with I^2^ values of 80% in RFS and 75% in PFS. To the best of our knowledge, this was the first meta-analysis of Ki-67 in bladder cancer. To determine the origins of the heterogeneity, we performed a meta-regression including publication year, region, HR estimation, and analysis results. Only analysis results were significantly associated with heterogeneity of studies reporting RFS. Although region might have accounted for part of the inter-study heterogeneity, analysis results was observed to significantly affect the relationship of Ki-67 expression and PFS.

As a proliferation-associated nuclear antigen, Ki-67 is expressed in all phases of the cell cycle except G_0_. The normal bladder uroephithelium has a very low proliferation rate [13], increased proliferation may signal recurrence rate, and high Ki-67 expression has a poor prognosis for patients with bladder cancer. Bladder tumors with Ki-67 expression have aggressive behaviors, such as multifocality, concomitant CIS, and increased EORCT risk scores, in addition to higher grade/stage [14, 15]. Because Ki-67 is a cellular proliferation marker, some studies claim that it is more closely related to the recurrence of NMIBC rather than progression to MIBC [14, 16]. Other studies reported that Ki-67 was related not only to recurrence but also to progression and survival [15, 17, 18]. Even though a consensus on the prognosis of Ki-67 expression has not been reached, this meta-analysis found that patients with high Ki-67 expression had significantly higher recurrence and progression rates than those with low expression. Even though the meta-analysis of DSS included only six studies and that of OS only two, patients with high Ki-67 expression had a significantly worse prognosis.

There were two notable study limitations. The first was study heterogeneity, which is common to meta-analyses of prognostic marker studies. Even though we applied strict inclusion and exclusion criteria to all study stages, and the selected studies included patient populations with similar T stage and grade, the variables evaluated study was different and diverse. Second, because of the strict selection criteria, we were not able to perform Begg tests as fewer than 10 studies were included in the DSS and OS meta-analysis. Consequently, while the analysis generated symmetrical inverted funnel plots, the results should be interpreted with care because of publication bias.

## MATERIALS AND METHODS

This meta-analysis was performed following the Preferred Reporting Items for Systematic Reviews and Meta-Analyses (PRISMA) guidelines [19].

### Search strategy

Embase, Scopus, and PubMed were searched for articles published in English to March 28, 2016 using the keywords “bladder cancer” and “Ki-67.” The titles and abstracts of the retrieved articles were reviewed independently by two authors (KK and CWJ) to minimize bias and to improve reliability. The reference lists of the retrieved articles were manually searched for potentially eligible studies that were not included in the initial database search. The full texts of the selected articles were independently screened by the same authors. Disagreements between the reviewers were resolved by consensus.

### Study selection

**Figure 3:**
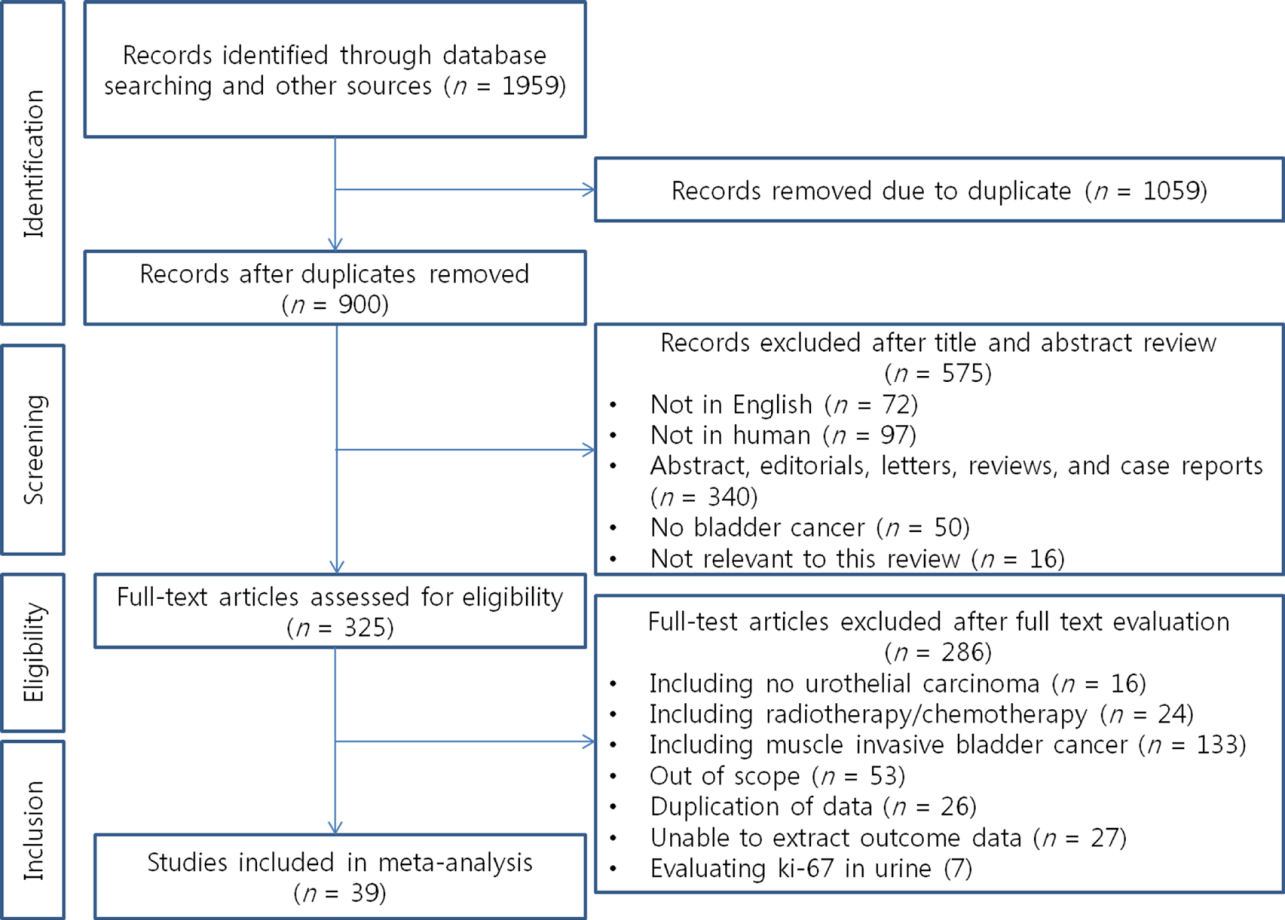
The PRISMA flow chart

The PRISMA flow chart of the systematic literature search and study selection is shown in Figure 3. The initial searches retrieved 1,959 articles. Of these, 1,059 were excluded as duplicate publications and an additional 575 were excluded after reviewing the abstracts. The full texts of the remaining 325 articles were reviewed, and an additional 286 articles that did not satisfy the inclusion criteria were excluded. A total of 39 articles including 5,229 patients, ranging from 32 to 605 per study were finally included in the analysis [6, 10, 11, 14–18, 20–50].

### Inclusion and exclusion criteria

Following the PRISMA guidelines, the study population, intervention, comparator, outcome, and study design (PICOS) were used to define study eligibility [19]. In this analysis, these were defined as *Population*, patients with NMIBC; *Intervention*: TURBT; *Comparator*, Ki-67 expression; *Outcome*, recurrence, progression, cancer-specific mortality, and any-cause mortality; *Study design*, univariate and/or multivariate Cox regression analysis. Strict, well-defined inclusion and exclusion criteria were intended to limit heterogeneity across studies and facilitate obtaining clinically meaningful results in this meta-analysis of prognostic marker studies [51]. The eligibility criteria were as follows: publication as an original article in English language; included human research subjects who were NMIBC patients and treated with TURBT; reported the histologic type as urothelial carcinoma (UC); evaluated Ki-67 expression in bladder cancer tissue by IHC; and investigated the association of Ki-67 expression level and survival outcomes. Eligible articles reported Kaplan–Meier/Cox regression-derived results of the prognostic value of Ki-67 on outcomes following the REporting recommendations for tumor MARKer prognostic studies (REMARK) guidelines for assessment of prognostic markers [52].

Studies were excluded if they were: letters, commentaries, case reports, reviews, or conference abstracts (because of limited data); studies conducted in animals or cell lines; studies using other than survival analyses.

If the same patient series was included in more than one publication, only the most informative or complete report was included to avoid duplication of the survival data. Two reviewers (CK and HHK) independently determined study eligibility. Discrepant opinions were resolved by discussion.

### End points

The primary outcome measures were RFS, PFS, DSS, and OS. Survival was defined as the time from TURBT to the last follow-up. In the meta-analysis, recurrence was the development of histologically confirmed UC on follow-up after complete tumor resection. Disease-specific death was any death because of bladder cancer in patients with documented metastatic or recurrent disease. Compared with the primary tumor, progression was defined in individual studies as development of a higher stage [6, 14]; development of a higher stage and/or grade [27, 31]; development of a higher stage and/or grade as well as development of regional or distant metastases [25]; development of a higher stage or metastasis [10, 16, 17, 33, 36, 37, 41], or development of a higher stage and muscle invasive cancer (≥ T2), distant metastasis, or death from bladder cancer [11]. Additional definitions of progression included development of MIBC (≥ T2) [34, 45, 47] and development of MIBC (≥ T2) and/or metastasis [15, 49, 50].

### Data extraction

Two reviewers (KK and JHK) extracted the study characteristics and outcome data, which were subsequently crosschecked to ensure their accuracy. Any discrepancies in extracting data were resolved by discussion. Authors of eligible studies were not contacted for additional data. The data retrieved following the REMARK guidelines were: the name of first author, country and year of publication, geographic location, study design, and recruitment period; the study population sample size, mean or median age, gender distribution, inclusion and exclusion criteria, treatment administered, endpoint definition, and follow-up period; tumor characteristics including stage, and grade; IHC data including cutoff value of positive expression, the antibodies used; adoption of a blinded evaluation method; and statistical data including survival curves, data including the total number of case and control participants, and HRs with confidence intervals (CIs). Discrepancies were resolved by discussion.

### Statistical analysis

The meta-analysis was carried out with Review Manager software (RevMan 5; The Nordic Cochrane Center, The Cochrane Collaboration, Copenhagen, Denmark) and R 2.13.0 (R Development Core Team, Vienna, Austria, http://www.R-project.org).

### Primary analysis

Study and pooled estimates were presented as forest plots. Survival outcome data were synthesized using the time-to-event HR as the operational measure. The method used to estimate the HR of each publication depended on the data provided. If HRs and the corresponding standard errors were not directly reported, then previously reported indirect methods were used to extract the logHR and variance because of the lack of previously published prognostic values [53–55]. A DerSimonian and Laird random effects model was used to obtain the summary HRs and 95% CIs.

### Assessment of heterogeneity

Heterogeneity of combined HRs was evaluated by the chi-square test and Higgins I-squared statistic. With the chi-square test, heterogeneity was significant when the *p*-value was < 0.05. I^2^ described the proportion of total variation in meta-analysis estimates that was caused by inter-study heterogeneity, rather than sampling error. It can take a value from 0% to 100%; increasing I^2^ values indicated increasing between-study heterogeneity. An I^2^ value above 50% was considered as having notable heterogeneity [56, 57], and if found, a subgroup meta-regression analysis was carried out to identify the source of the heterogeneity.

### Publication bias

Publication bias was evaluated with funnel plots. In the absence of bias, the plots should resemble a symmetrical, inverted funnel and in the presence of bias, they should appear skewed and asymmetrical [57]. If more than 10 studies were included in the meta-analysis, then the Begg rank correlation test was also used to evaluate publication bias [58]. Bias was assumed if the *p*-value was < 0.05.

### Role of the funding source

The funding source had no role in the study design, the collection, analysis, and interpretation of data, or the writing of the report. The corresponding author had full access to all data and had final responsibility to submit the paper for publication.

## Abbreviations

MIBC: Muscle invasive bladder cancer
NMIBC: Non-muscle invasive bladder cancer
TURBT: Transurethral resection of bladder tumorl
CIS: Carcinoma *in situ*
IHC: Immunohistochemistry
RFS: Recurrence-free survival
PFS: Progression-free survival
DSS: Disease-specific survival
OS: Overall survival
HRs: Hazard ratios
PRISMA: Preferred Reporting Items for Systematic Reviews and Meta-Analyses
PICOS: Population, intervention, comparator, outcome, and study
UC: Urothelial carcinoma
REMARK: REporting recommendations for tumor MARKer prognostic studies
CIs: Confidence intervals
